# Direct detection of nasal *Staphylococcus aureus* carriage via helicase-dependent isothermal amplification and chip hybridization

**DOI:** 10.1186/1756-0500-5-430

**Published:** 2012-08-11

**Authors:** Georges C Frech, Denton Munns, Robert D Jenison, Brian J Hicke

**Affiliations:** 1 Great Basin Corporation, 2441 South 3850 West, Salt Lake City, UT, 84120, USA

**Keywords:** *Staphylococcus aureus*, Nasal carriage, Molecular diagnostic, Helicase-dependent amplification

## Abstract

**Background:**

The bacterium *Staphylococcus aureus* constitutes one of the most important causes of nosocomial infections. One out of every three individuals naturally carries *S. aureus* in their anterior nares, and nasal carriage is associated with a significantly higher infection rate in hospital settings. Nasal carriage can be either persistent or intermittent, and it is the persistent carriers who, as a group, are at the highest risk of infection and who have the highest nasal *S. aureus* cell counts. Prophylactic decolonization of *S. aureus* from patients’ noses is known to reduce the incidence of postsurgical infections, and there is a clear rationale for rapid identification of nasal *S. aureus* carriers among hospital patients.

**Findings:**

A molecular diagnostic assay was developed which is based on helicase-dependent target amplification and amplicon detection by chip hybridization to a chip surface, producing a visible readout. Nasal swabs from 70 subjects were used to compare the molecular assay against culturing on “CHROMagar Staph aureus” agar plates. The overall relative sensitivity was 89%, and the relative specificity was 94%. The sensitivity rose to 100% when excluding low-count subjects (<100 *S. aureus* colony-forming units per swab).

**Conclusions:**

This molecular assay is much faster than direct culture and has sensitivity that is appropriate for identification of high-count (>100 *S. aureus* colony-forming units per swab) nasal *S. aureus* carriers who are at greatest risk for nosocomial infections.

## Findings

### Background

Nosocomial bacterial infections are an important cause of morbidity and mortality, attributable for approximately 100,000 deaths annually in the United States [1]. The Gram-positive bacterial pathogen *Staphylococcus aureus* is causally involved in a significant fraction of these infections, with about 0.8% of all US hospital inpatients suffering from a *S. aureus* infection [2]. The anterior nares represent the primary ecological reservoir for *S. aureus* in humans, with one in three individuals being carriers [3]. Three different nasal carriage patterns have been discerned, with approximately 20% of the population being persistent carriers, 30-60% intermittent carriers, and the remainder being non-carriers [4]. The quantity of *S. aureus* colony-forming units (CFU) that can be recovered from swabs obtained from carriers’ noses varies widely, from single-digits to millions [5,6], and there is a strong association between high cell count and persistent carriage [7-9]. Based on various levels of evidence, it has been suggested that persistent carriers represent a separate group that is distinct from intermittent and non-carriers [8,10].

Colonization of the human nose by *S. aureus* represents a commensal relationship, and carriage is inconsequential to the healthy human host in every-day life. However, *S. aureus* nasal carriage translates into a three to four fold higher infection rate in hospital settings compared to non-carriers [11-14]. In one population of dialysis patients, persistent nasal *S. aureus* carriers carried a 3.4 times higher risk than intermittent carriers [13]. Prospective and retrospective studies revealed that the majority of nosocomially infected nasal carriers suffer from a *S. aureus* strain that is clonally identical to the commensal strain carried in their nose, thereby strongly implicating an endogenous origin [11,14-16].

Topical intranasal mupirocin application provides an effective and safe option for *S. aureus* decolonization and, when used prophylactically, reduces the incidence of postsurgical infections [17]. The strongest evidence so far was provided by a double-blind, placebo-controlled, multicenter trial that used randomized patient groups from multiple hospitals in the Netherlands [16]. The results revealed a 2.4-fold reduction in risk of surgical-site *S. aureus* infections for the treatment group. The authors concluded that rapid identification of nasal *S. aureus* carriage at the time of hospital admission speeds decolonization and is a critical factor in reducing hospital-associated *S. aureus* infections. Due to the reported emergence of mupirocin resistance, it is prudent to restrict nasal mupirocin application only to patients who are likely to benefit from it [18]. This provides a strong incentive for hospital-based nasal *S. aureus* screening of patients who will be undergoing an invasive medical procedure, by using a molecular diagnostic test for rapid identification of nasal carrier status and initiation of *S. aureus* decolonization without delay.

Commercially available nasal screening tests generally use either polymerase chain reaction (PCR)-based or microbiological culture-based methods. PCR-based molecular diagnostic tests provide much faster turnaround relative to the lower cost culture-based methods, and the benefit of rapid turnaround has been well established [19]. The commercial PCR assay designs in use identify methicillin-resistant *S. aureus* (MRSA) [20-23], and they do so by targeting a mobile staphylococcal cassette chromosome (SCC) element referred to as SCCmec [24]. This assay design causes false positive rates that significantly impact MRSA screening efficacy [25-27], in particular in geographic regions where MRSA prevalence is low or on the decrease, and it can also cause false negatives [28,29]. We have developed an alternative molecular detection method that targets *S. aureus*-specific sequences in the thermonuclease (nuc) gene [30,31]. The assay system recapitulates the rapid turnaround time of PCR but at lowered cost, using isothermal amplification coupled to chip-based detection followed by digital camera capture of the chip image [32].

### Subjects, materials and methods

#### Subjects and nasal swab collection

Subjects who volunteered to provide nasal swabs for this study were healthy individuals, 39 male and 31 female. Subjects provided written informed consent for their nasal swab samples to be utilized in this study, which was approved by the Great Basin Corporation internal Ethics Committee. All samples were de-identified. Each sterile media-free Double Swab (BD BBL CultureSwabs 220135) was pre-moistened with 75 uL sterile phosphate buffered saline (PBS) and pre-scored for subsequent facilitated detachment of the polyester tip from its stem. Volunteers were instructed to insert the Double Swab into one of their nostrils and rotate it for at least 15 seconds while scrubbing the surface of the anterior nare, and then repeat the same procedure for the second nostril. The swabs were kept at ambient temperature and processed no later than 2 hours after sampling. Swab A was used for microbiological examination and swab B was processed for molecular analysis.

#### Direct culture

The head of swab A was detached from its stem, transferred into a tube containing 200 uL 10 mM Tris (pH 8.8), 10 mM NaCl (TN-Buffer), and vortexed for 30 seconds at maximum setting. This yielded a recoverable volume of approximately 160 uL nasal mucus suspension, and 100 uL thereof was directly plated onto a “BBL CHROMagar Staph aureus” agar plate (Becton Dickenson) which was then incubated at 37°C for 18–20 hours. The samples that gave rise to mauve colonies on plates, indicative of *S. aureus*, were scored as positive by direct culture. The number of mauve colonies per plate was counted exactly (if <1000) or estimated (if >1000), and *S. aureus* CFU counts per swab were extrapolated by multiplying with a factor of 1.6.

#### Preparation of bacterial DNA from nasal swabs

The head of swab B was detached from its stem, placed into 500 uL TN-Buffer, and vortexed for 30 seconds at maximum setting. The sample was then centrifuged for 10 minutes at 14,000 x g at ambient temperature. The supernatant was discarded and the pellet re-suspended in 100 uL TN-Buffer containing 0.5 U/uL achromopeptidase (Wako Chemicals, Richmond, VA). The mixture was incubated for 15 minutes at 37°C, followed by 5 minutes at 98°C. Two 5 uL aliquots were used in replicate Helicase-dependent amplification reactions, and the rest was stored frozen at −20°C.

#### Asymmetric helicase-dependent amplification (HDA)

Amplification reactions were set up according to conditions as provided by the IsoAmp II Universal tHDA Kit (BioHelix, Beverly, MA). For each individual HDA reaction, 5 uL of nasal mucus lysate was first mixed with 15 μL of Dilution Buffer, giving rise to concentrations of 20 mM Tris–HCl, pH 8.8, 10 mM KCl, 7.7 mM MgSO_4_, 40 mM NaCl, 5 mg/mL BSA, and 0.02% Tween 20. This was then added to 20 uL 2x HDA-Mix {20 mM Tris–HCl, pH 8.8, 40 mM NaCl, 0.02% Triton X-100, 0.4x EvaGreen (Biotium, Hayward, CA), 6.8 mM dATP, 0.8 mM dCTP, 0.8 mM dGTP, 0.8 mM dTTP, 10 ng/uL uvrD Thermostable DNA Helicase, 1.6 U/uL GST Polymerase Large Fragment, 4 ng/uL ET SSB (Extreme Thermostable Single-Stranded DNA Binding Protein), and diluted RNase H2 (Great Basin Corp), 600 nM Forward primer, and 800 nM biotinylated Reverse primer (5’ Biotin-TEG)} and mixed thoroughly in a well of a LightCycler 480 Multiwell Plate 96 (Roche Diagnostics, Indianapolis, IN). The multiwell plate was transferred into a LightCycler 480 instrument and incubated for 60 minutes using an isothermal temperature profile set to 65°C.

Primers were ordered from IDT, and each primer contained a single ribo-nucleotide close to the 3’ end as well as a 3’ blocking group, creating “hot start-like” conditions. Deblocking requires that primers be annealed to the target DNA sequence, resulting in cleavage by RNase H2 at temperatures above 50°C, followed by polymerase extension. Primer artifact is suppressed because the RNase H2 is inactive at low temperature. The nucleotide sequences of the amplification primers were 5’ –TGGTAGAAAATGCAAAGAAAATTGAAGTC[rG]AGTT-3’ for the Forward primer and 5’- TCCATCAGCATAAATATACGCTAAGCCA[rC]GTCC-3’ for the Reverse primer, giving rise to an amplification product size of 95 base pairs.

#### Chip preparation and hybridization

Crystalline silicon wafers were coated with the polymer amino functional T-structure poly-dimethylsiloxane (TSPS, United Chemical Technologies, Bristol, PA) and cured at 150°C for 24 hours. The TSPS coated wafer was further prepared by soaking in a 50 mg/L solution of poly (lys-phe) in 1x phosphate-buffered saline (PBS, pH 6) containing 2 M NaCl overnight at room temperature. Next, the poly (lys-phe) coated wafer was washed and soaked in 10 μM succimidyl-4-formyl benzoate (SFB, Sigma-Aldrich, St. Louis, MO) for 2 hours at room temperature, washed thoroughly with water, dried with a stream of nitrogen, and stored at room temperature.

Capture probes were synthesized by IDT and contain an internal 18-atom hexa-ethyleneglycol spacer connected to a reactive 5’-moiety (I-Linker) that couples to the aldehyde-functionalized chip surface. Probes in Spotting Buffer (0.1 M phosphate buffer pH 8.0, 10% glycerol) were spotted (75 nL) on the surface of the SFB-coated silicon wafer. A biotin-labeled [dA]_18_ detection control (DC) probe was spotted at 50 nM, and a hybridization control (HC) capture probe was also spotted at 50 nM (complementary to biotinylated detection probe present in bybridization buffer). To orient chips for subsequent processing a fiducial marker (carboxylated polystyrene microspheres) was also printed. The *S. aureus nuc* amplicon-specific capture probe was spotted at 400 nM concentration, and its DNA sequence was 5’-GACAAAGGTCAAAGAACTGA-3’. After incubating for 2 hours, the wafers were washed with 0.1% SDS, dried, and scribed into individual 6.7 mm square chips before use.

For chip hybridization, 20 μL of HDA amplicon was added to 80 μL of hybridization buffer (5x SSC, 0.05% Tween 20, 0.5% alkaline-treated casein (ATC), 1 nM hybridization control (HC) biotinylated detection probe) and pre-warmed in a heat block at 65°C for 5 minutes. The hybridization mixture was then transferred onto chips in a 96-well plate that had been prewarmed to 53°C, and hybridization was allowed to proceed for 5 minutes at 53°C. Solution was removed, and the chips were washed 3 times with WashBuffer-A (0.1x SSC, 0.1% SDS), then washed 3 times with WashBuffer-B (0.1x SSC, 0.05% Tween 20), before adding 100 μL of conjugate solution (1 μg/mL anti biotin antibody/HRP in 5x SSC, 10% fetal calf serum, 0.5% ATC) and incubating at room temperature for 4 minutes. Chips were then washed 3 times with WashBuffer-B before addition of 100 μL Membrane 3,3',5,5'-Tetramethylbenzidine (TMB, BioFX Laboratories, Eden Prairie, MN) to the chip surface and incubation at room temperature for 4 minutes. Chips were washed with water and methanol, air-dried, and then imaged.

#### Discrepancy resolution analyses

Nasal swab samples that gave rise to a negative result by direct culture but a positive result by the molecular assay were followed up by a repeat of the HDA and chip hybridization, using fresh aliquots of the original nasal swab lysates. Furthermore, HDA discordant positives were followed up by PCR amplification (Roche LightCycler 480) using the *nuc* gene amplification primers published by Brakstad and coworkers [31]. A product band of the correct size was verified by polyacrylamide gel electrophoresis. In addition, volunteers whose nasal swabs produced molecular assay discordant positives were asked to provide follow-up nasal Double Swabs, and these were processed as described above for direct culture as well as molecular assay analysis.

Volunteers whose nasal swabs gave rise to a positive result by direct culture but a negative result by the molecular assay, were asked to provide a follow-up nasal swab a few weeks later. These follow-up swabs were processed by direct culturing as described above.

### Results and discussion

#### Analytical assay performance

The molecular diagnostic assay described in this study is based on isothermal helicase-dependent amplification (HDA) [33] of *Staphylococcus aureus*-specific DNA sequences, derived from the thermonuclease gene *nuc*, followed by hybridization of the biotinylated amplification product to a *nuc*-specific capture probe immobilized on silicon chips. To define assay sensitivity, pooled nasal mucus was spiked with dilutions of *S. aureus* cells, and the chip could detect as few as 2 CFU per HDA reaction, which extrapolates to 40 CFU per swab (data not shown). This indicates that the protocol developed for this study efficiently enriched and lysed *S. aureus* cells present in nasal mucus, making the bacterial genomic DNA available for amplification. A panel of 21 *S. aureus* strains was then used to test reactivity of the *nuc* gene HDA primers and hybridization probe. All 21 strains gave rise to positive HDA and chip hybridization signals (Table 1). To verify that the assay detects only *Staphylococcus aureus*, a panel of prokaryotic and eukaryotic organisms was examined. No positive *nuc* amplicon signal was observed for the 8 other staphylococcal species or the 17 additional bacterial and eukaryotic organisms that were tested (Table 2). These results are consistent with previous studies establishing that the *nuc* gene contains *S. aureus*-specific sequences [30,31] and with a recently published study in which the *nuc* gene was present in 1781 of 1783 *S. aureus* isolates (99.9% sensitivity) [34].

**Table 1:** ***Staphylococcus aureus *strains tested for sensitivity of amplification primer set and detection probe**

**Organism**	**ATCC number**	**Other designations**	**Molecular assay**
*S. aureus* subsp. *aureus* Rosenbach	6538	FDA 209	positive
*S. aureus* subsp. *aureus* Rosenbach	14993	PCI 1217 [21 J]	positive
*S. aureus* subsp. *aureus* Rosenbach	25923	Seattle 1945	positive
*S. aureus* subsp. *aureus* Rosenbach	33591	328	positive
*S. aureus* subsp. *aureus* Rosenbach	33592	1063	positive
*S. aureus* subsp. *aureus* Rosenbach	43300	F-182	positive
*S. aureus* subsp. *aureus* Rosenbach	BAA-1720		positive
*S. aureus* Rosenbach	BAA-1749	96:308	positive
*S. aureus* Rosenbach	BAA-1764	7031	positive
*S. aureus* Rosenbach	BAA-1765	102-04	positive
*S. aureus* subsp. *aureus* Rosenbach	BAA-42	HDE288	positive
*S. aureus*		ANS46	positive
*S. aureus* subsp. *aureus* COL		STAAC, 93062	positive
*S. aureus*		HDG2	positive
*S. aureus*		MA14	positive
*S. aureus*		MA15	positive
*S. aureus*		MA6	positive
*S. aureus*		MA8	positive
*S. aureus*		MSH7	positive
*S. aureus* subsp. *aureus* MW2		STAAW, 196620	positive
*S. aureus*		WIS	positive

Isolated microbial colonies were suspended in TN-Buffer, lysed by incubation with achromopeptidase followed by boiling, and the equivalent of in the order of 10^6^ cells was added to each HDA molecular assay reaction.

**Table 2:** Species tested for specificity of amplification primer set and detection probe

**Organism**	**Source of isolate**	**Characteristics**	**Molecular assay**
*S. schleiferi*	ATCC # 43808	Coagulase-positive	negative
*S. capitis*	ATCC # 35661	Coagulase-negative	negative
*S. epidermidis*	ATCC # 12228	Coagulase-negative	negative
*S. haemolyticus*	ATCC # 29970	Coagulase-negative	negative
*S. hominis*	ATCC # 700236	Coagulase-negative	negative
*S. lugdunensis*	ATCC # 43809	Coagulase-negative	negative
*S. saprophyticus*	ATCC # 15305	Coagulase-negative	negative
*S. succinus*	ATCC # 700337	Coagulase-negative	negative
*Bacillus subtilis*	ATCC # 23859	gram-positive	negative
*Clostridium difficile*	ATCC # BAA-1382	gram-positive	negative
*Enterococcus faecalis*	ATCC # 700802	gram-positive	negative
*Enterococcus faecium*	ATCC # 51559	gram-positive	negative
*Micrococcus luteus*	ATCC # 10240	gram-positive	negative
*Mycobacterium abscessus*	ATCC # 19977	gram-positive	negative
*Streptococcus agalactiae*	ATCC # 13813	gram-positive	negative
*Streptococcus pneumoniae*	ATCC # 6308	gram-positive	negative
*Acinetobacter baumannii*	ATCC # 17978	gram-negative	negative
*Citrobacter freundii*	ATCC # 8090	gram-negative	negative
*Escherichia coli*	ATCC # 4157	gram-negative	negative
*Klebsiella pneumoniae*	ATCC # 13883	gram-negative	negative
*Neisseria gonorrhoeae*	ATCC # 53420	gram-negative	negative
*Pseudomonas putida*	ATCC # 47054	gram-negative	negative
*Candida albicans*	ATCC # 18804		negative
*Saccharomyces cerevisiae*	Strain S288C		negative
*Homo sapiens*	Roche Cat. #11691112001		negative

Isolated microbial colonies were suspended in TN-Buffer (see Materials and Methods section), lysed by incubation with achromopeptidase and/or boiling, and the equivalent of in the order of 10^6^ cells (or 20 ng purified yeast DNA or 80 ng human DNA) was added to each HDA molecular assay reaction.

#### *S. aureus*-positive samples

A total of 70 volunteers were enrolled for this nasal *S. aureus* detection study. Of these, 36 individuals were culture-positive on “CHROMagar Staph aureus” plates, with a range between 3 and more than 100,000 CFU per swab (Table 3). In comparison to direct culture, the molecular diagnostic assay had a relative sensitivity of 89% (Table 4). Using a cutoff of 100 *S. aureus* CFU per swab, the samples were stratified into “high-count” and “low-count” groups. This cutoff was selected originally based on data provided in Figure 2 from the article by Nouwen et al. [7], which indicated that swabs with more than 100 CFU are significantly more likely to originate from persistent carriers than from intermittent carriers. More recently published results revealed that 20/21 samples from persistent carriers were associated with nasal *S. aureus* loads of >100 CFU per swab, and 13/14 samples from non-persistent carriers were associated with loads of ≤100 CFU [9], further validating our selection of this cutoff value. Of the 36 nasal *S. aureus* carriers identified in this study, 29 were classified into the high-count group. All of these 29 individuals were positive by the molecular assay (Table 3), and for this subgroup the concordance between the molecular assay and direct culture was 100% (Table 5). Seven samples had fewer than 100 *S. aureus* CFU per swab (Table 3), four of which were negative by the molecular assay. This is not surprising since these samples contained *S. aureus* CFU numbers near or below the lower limit of detection. Two discrepant samples were confirmed as *nuc*-negative (Table 3) by PCR analysis using previously published *nuc* gene primers [31].

**Table 3:** Nasal swab samples giving rise to mauve colonies on “CHROMagar S. aureus” agar plates

**#**	**Initial Swab: CFU/Swab**	**Molecular assay**	**Follow-up PCR**	**Follow-up Swab: CFU/Swab**
**1-27**	**>100,000 to >2,000**	**All Positive**		
**28**	**1152**	**Positive**		
**29**	**387**	**Positive**		
30	38	Positive		
31	27	Negative	ND	594
32	8	Positive		
33	6	Negative	ND	0
34	5	Positive		
35	3	Negative	Negative	ND
36	3	Negative	Negative	0

Samples are sorted by CFU/Swab. The 29 samples giving rise to >100 mauve colonies per plate were classified as “high-count” and are indicated in bold.

CFU: Colony Forming Units. ND: Not Done.

**Table 4:** Comparison of the molecular assay results to the direct culture method

		**Direct culture**		**Total**
		**Positive**	**Negative**	
**Molecular Assay**	Positive	32	2	34
	Negative	4	32	36
	Total	36	34	70
**Sensitivity:**	89%			
**Specificity:**	94%			

**Table 5:** **Stratification of *S. aureus *-positive samples: high-count group only (>100 CFU/swab)**

	**Number of samples**	
	**Positives**	**Negatives**
**Direct Culture**	29	0
**Molecular Assay**	29	0
**Concordance:**	100%	

Individuals who carry low numbers of *S. aureus* cells are more likely to be intermittent rather than persistent nasal carriers [7-9]. Three volunteers whose nasal swab samples gave rise to discrepant results (#31, #33, #36, Table 3) were able to provide a follow-up swab a few weeks later. Two of the three follow-up swabs turned up negative on direct culture, classifying these two volunteers as intermittent *S. aureus* carriers (#33, #36, Table 3).

#### *S. aureus*-negative samples

Of the 70 volunteers, 34 were culture-negative. Two of these 34 samples gave rise to a positive result by the molecular assay, which translates into a relative specificity of 94% (Table 4). Both samples remained positive upon repeated molecular analysis. Follow-up Double Swabs were subsequently obtained from the two volunteers. Swabs from both individuals were again molecular assay-positive, and individual I was again culture-negative while individual II yielded 3 CFU, indicating that individual II was a low-count carrier. The samples from both volunteers were confirmed as *nuc*-positive by PCR analysis [31].

Resolution of the discrepancies between the identification of *S. aureus* by *nuc* gene amplification versus direct microbiological culture will require further analyses. While the microbiological method chosen for this study, direct culturing on “CHROMagar Staph aureus” chromogenic medium, is characterized by its simplicity as well as excellent specificity, the sensitivity of this method is not 100%. Among 310 *S. aureus*-positive clinical specimens, CHROMagar Staph aureus was 95.5% sensitive [35]. Therefore, one would expect one or two of the 34 negative samples in the present study to be falsely negative. Alternatively, it is conceivable that swabs obtained from certain low-count individuals may contain mostly dead cells that would go undetected by culture, since this method relies upon the presence of live cells. Discrepancy resolution might be achieved by the amplification and sequencing of the rpoB gene which allows for accurate differentiation of staphylococcal isolates at the species and subspecies level [36]. However, since the nasal environment is known to contain a mixture of multiple bacterial species [37], sequencing of amplification products obtained from nasal swab lysates would require sequence analysis of large numbers of clones.

#### Prevalence of *S. aureus* nasal carriage

The fraction of volunteers in this study who were positive for nasal carriage of *S. aureus* (36-38/70; 51-54%) is higher than the 32% prevalence that was reported by the large (~10,000 subjects) US National Health and Nutrition Examination Survey conducted between 2001 and 2002 [3]. Notably, nasal *S. aureus* carriage clusters in families [38], and the higher prevalence observed in the present study could be explained by the fact that the volunteer group did not represent a random population sample but contained a significant number of biological relatives. Furthermore, the volunteer group for this study contained more males than females, and the *S. aureus* nasal carriage prevalence is higher among males than females [3]. In addition, the dry climate in Utah, where this study was conducted, is associated with higher rates of nose bleeding which is correlated with nasal *S. aureus* carriage [39] and appears to be directly mediated by the presence of hemoglobin [40].

#### Conclusions

The molecular diagnostic assay described in this study combines helicase-dependent isothermal amplification of a *S. aureus* species-specific DNA sequence out of nasal swab lysate with chip-based detection by hybridization and an eye-visible readout. In the present study involving nasal swabs from 70 volunteers, this molecular assay showed 100% sensitivity in identifying those individuals who are high-count nasal carriers of *S. aureus* (>100 CFU per swab). It is these individuals who have increased risk of infection after invasive procedures in hospitals, and therefore need to be rapidly identified and de-colonized prior to an invasive procedure. The assay concept presented here lends itself to incorporation into an automated molecular diagnostic platform for rapid identification of nasal *S. aureus* carriers in hospital settings.

Updated commercial PCR strategies can incorporate mecA gene amplification to mitigate the problem of false positive results in so-called "empty cassette" strains that result from incomplete SCCmec cassette excision (Arbefeville SS et al 2012. J Clin Micro 49:2996-2999).

## Competing interests

All authors were employed by the company Great Basin Corporation.

## Authors’ contributions

GCF wrote the manuscript, participated in the design of the study, acquisition of data, and directed the analysis and interpretation of the data. DM was responsible for data acquisition and participated in the analysis and interpretation of the data. RDJ originated the assay concept and critically revised the manuscript. BJH participated in the design and conception of the study, and critically revised the manuscript. All authors read and approved the final manuscript.
